# Time-Restricted Eating, Cardiometabolic Health in Obesity and The Optimal Length of the Eating Window

**DOI:** 10.1007/s13668-026-00742-8

**Published:** 2026-03-09

**Authors:** Bianca Monteiro Silva, Gabriela Geraldo Benzoni, Marcela Coffacci de Lima Viliod, Caroline Fogagnolo, Gabriela Ferreira Abud, Gabriela Ueta Ortiz, Ivo Vieira de Sousa Neto, Ana Cláudia Rossini-Venturini, Sofia Germano Travieso, Ellen Cristini de Freitas

**Affiliations:** 1 Department of Health Sciences, Ribeirao Preto Medical School, University of Sao Paulo (FMRP/USP), Ribeirao Preto, Sao Paulo, Brazil; 2 Laboratory of Exercise, Physiology and Metabolism, School of Physical Education and Sport of Ribeirao Preto, University of Sao Paulo (EEFERP/USP), Ribeirao Preto, Sao Paulo, Brazil; 3 School of Physical Education and Sport of Ribeirao Preto, University of São Paulo (EEFERP/USP), Ribeirao Preto, Sao Paulo, Brazil

**Keywords:** Time-Restricted eating, Eating windows, Cardiometabolic health, Obesity, Circadian rhythms

## Abstract

**Purpose of Review:**

This narrative review aims to evaluate how the duration of eating windows influences the cardiometabolic effects of Time-Restricted Eating (TRE) protocols among individuals with obesity. In the context of the global obesity epidemic, TRE has emerged as a promising dietary approach, alternative to conventional energy restriction, capable of improving cardiometabolic health by aligning food intake with circadian rhythms. However, due to the heterogeneity across TRE protocols, the impact of eating windows length on these outcomes remains uncertain. Therefore, this review comprehensively analyzes clinical trials assessing cardiometabolic responses across various durations of eating windows to identify which protocol may provide the greatest benefits.

**Recent Findings:**

Short eating windows (4–6 h), although associated with promising metabolic outcomes, often exhibit poor adherence due to increased hunger, fatigue and social constraints, limiting their long-term viability. Moderate eating windows (8–10 h) appear to offer the optimal balance between metabolic benefits, minimal adverse effects and adherence. Long eating windows (12–14 h), despite higher adherence, provide limited metabolic outcomes and are frequently associated with circadian misalignment, potentially increasing the risk of obesity and related metabolic disorders. All the data mentioned were obtained from clinical trials.

**Summary:**

TRE has evolved as a promising strategy to enhance cardiometabolic health, especially in individuals with obesity. Eating windows of 8-10h appear to represent the most sustainable TRE protocol, balancing metabolic efficacy with adherence. Nevertheless, further well-designed, long-term randomized controlled trials incorporating chrononutritional assessments are required to establish the optimal eating window for maximizing health outcomes in obesity.

## Introduction

Obesity is a chronic, complex and multifactorial disease that has been recognized as one of the most critical public health challenges of the 21st century, given its increasing global prevalence and strong association with morbidity and mortality [1]. According to recent estimates from the World Health Organization (WHO), approximately 2.5 billion adults are currently living with overweight, of whom 890 million are classified as having obesity [2]. These data highlight the substantial burden of obesity on healthcare systems and the national economies worldwide [3].

Obesity is characterized by the excessive accumulation of adipose tissue, which may occur with or without alterations in its distribution or function, and results from a dynamic interaction of genetic, environmental, behavioral and social factors [4]. When associated with adipose tissue dysfunction, this abnormal fat mass expansion contributes to the establishment of a low-grade systemic inflammatory state, which plays a central role in increasing the risk of several non-communicable chronic diseases, including type 2 diabetes mellitus (T2DM), dyslipidemia, hypertension, cardiovascular disease, and certain types of cancer [5].

Although excessive energy intake (EI) combined with reduced physical activity remains the primary driver of obesity and its comorbidities, recent advances in metabolic research have underscored the importance of several factors in its pathophysiology, such as insufficient sleep, irregular eating patterns and circadian misalignment [6]. Disrupted or insufficient sleep has been shown to alter energy expenditure and appetite-related hormones, beyond promoting irregular feeding behavior, thereby contributing to the development of obesity and metabolic dysfunction. Conversely, obesity can further aggravate sleep disturbances, destabilize eating patterns and impair endocrine homeostasis, establishing a bidirectional interplay that reinforces its chronicity [7]. Notably, both insufficient sleep and irregular meal timing contribute to circadian misalignment, a condition that has been frequently associated with increased susceptibility of adverse metabolic outcomes, such as obesity, cardiovascular disease, T2DM and dyslipidemia [7].

Circadian rhythms are endogenous, approximately 24h cycles that regulate a wide range of physiological processes, including hormone secretion, energy metabolism and feeding behavior, in anticipation of and in response to predictable daily environmental changes [8]. These rhythms are orchestrated by a central clock located in the suprachiasmatic nucleus (SCN) of the hypothalamus, primarily regulated by the light/dark cycle, and by peripheral clocks, which are entrained by external cues (referred to as “zeitgebers”) such as feeding patterns, sound stimuli, ambient temperature and physical activity [9].

Disruption of circadian alignment, commonly observed in shift workers, individuals with disturbed sleep patterns, and those exposed to artificial light at night, has been increasingly linked with impairments in metabolic homeostasis. These include reduced insulin sensitivity, impaired glucose tolerance, increased waist circumference, dysregulation of appetite-regulating hormones, elevated triglycerides levels and decreased high-density lipoprotein (HDL) cholesterol [6, 8–11].

Findings from animal and human studies indicate that the timing of food intake is a key circadian disruptor. Altered eating patterns, such as late-night eating, may exacerbate weight gain and increase the risk of metabolic disorders [8–10]. In this context, evidence from clinical and experimental studies support Time-Restricted Eating (TRE) as a non-pharmacological dietary intervention that seeks to align food intake with circadian biology, thereby improving several metabolic parameters even in the absence of weight loss [10, 12, 13]. TRE (for human studies) or Time-Restricted Feeding (TRF, in animal studies) is a dietary approach in which food intake is confined to a fixed number of hours per day, with fasting during the remaining period, without necessarily modifying total EI, as commonly observed in energy restriction (ER) diets, or overall diet quality [14].

Despite the growing body of evidence supporting TRE as a promising dietary approach for weight management and metabolic health, a critical question remains regarding the optimal eating window. The substantial heterogeneity across TRE protocols, encompassing eating periods of 4–12 h initiated at different times of the day (early, mid or late), as well as interindividual differences in metabolic responses influenced by factors such as chronotype and comorbidities poses a critical challenge to the establishment of standardized recommendations or the identification of the most metabolically favorable TRE schedule [15–18]. Moreover, most clinical trials have been of relatively short duration (typically 4 to 12 weeks), limiting the understanding of the long-term efficacy and safety of TRE in populations with obesity [19–23].

Therefore, this narrative review aims to synthesize the current evidence on the cardiometabolic effects of different durations of eating windows in TRE protocols among individuals with obesity, in order to assess whether the length of eating windows per se influences cardiometabolic outcomes. We first provide the historical framework, then focus on revisiting the advances in circadian rhythms’ physiology and molecular mechanisms inherent to TRE. In the second part, we critically examine how different eating window lengths may influence cardiometabolic outcomes including glycemic and lipid profile, blood pressure, oxidative and inflammatory markers and explore whether specific TRE schedules may be more effective for this condition. Additionally, this work aims to provide essential insights for researchers and practitioners to guide future investigations in chronotherapy, providing clinically useful information that helps design effective nutritional interventions.

## Physiological Basis of Circadian Rhythms

### Origin, Evolutionary Role and Classification of Biological Rhythms

According to chronobiology, throughout evolution, living organisms developed adaptive physiological mechanisms in response to selective pressures imposed by environmental phenomena, such as the alternation of light and darkness and fluctuations in temperature. These adaptations enabled organisms to anticipate and synchronize their biological functions with the environment changes daily, monthly, or yearly, thereby enhancing survival and ensuring optimal physiological functioning [24].

The term biological rhythms refers to cyclical patterns of biological activity that repeat at regular intervals. These rhythms are broadly categorized into three main types based on their periodicity. First, circadian rhythms, approximately 24h cycles. Second, ultradian rhythms, cycles with a period shorter than 24h and, therefore, occurring more than once per day (e.g., hormonal pulsatility, sleep cycles); and third infradian rhythms, cycles with a period longer than 24h and, therefore, occur less frequently than once per day, such as menstrual cycles or seasonal behaviors [24].

Among these, circadian rhythms are the most extensively studied due to their essential role in maintaining physiological processes and their strong association with the pathophysiology of several chronic diseases, including obesity, diabetes, cardiovascular disorders and cancer [25]. Given their central role in physiology and disease, circadian rhythms are orchestrated by a hierarchical system of central and peripheral clocks, as discussed in the following section.

### Definition and Regulation of Circadian Rhythms

Circadian rhythms are endogenous biological processes that exhibit a repeating pattern of approximately 24h which enable organisms to synchronize a broad spectrum of physiological and behavioral processes with external conditions. Through this synchronization, circadian regulation enhances metabolic, hormonal and cognitive functions and the body’s overall homeostasis [26].

The regulation of circadian rhythms is orchestrated by a hierarchical timekeeping system centered in the SCN of the hypothalamus. Acting as the master circadian pacemaker, the SCN governs rhythmicity across multiple levels of biological organization, ranging from the molecular regulation of gene expression to the coordinated functioning of peripheral organs. In addition to the central pacemaker, circadian regulation depends on peripheral clocks, which are self-sustained molecular oscillators present in virtually all organs and tissues, coordinated by the SCN and that drive tissue-specific rhythmicity across a wide array of physiological processes [27].

The central clock is synchronized primarily by light, detected by photosensitive retinal ganglion cells (pRGCs) containing melanopsin, which transmit photic information to the SCN via the retinohypothalamic tract (RHT). Upon receiving light input, the SCN conveys temporal signals to peripheral oscillators through neural, hormonal, and behavioral pathways, thereby aligning internal physiological processes with the external light-dark cycle [10, 28].

Although light is considered the predominant external cue for circadian synchronization, other environmental stimuli, known as zeitgebers (from the German term for “time giver”), also play critical roles in the regulation of biological clocks. These include ambient temperature, physical activity, feeding-fasting patterns, and, to a lesser extent, sound exposure [9]. At the molecular level, both central and peripheral clocks are governed by transcriptional-translational feedback loops (TTFLs), which constitute the core machinery of circadian regulation, as described in the following section.

### Molecular Mechanisms of the Circadian Clock: TTFLs

**Fig. 1:**
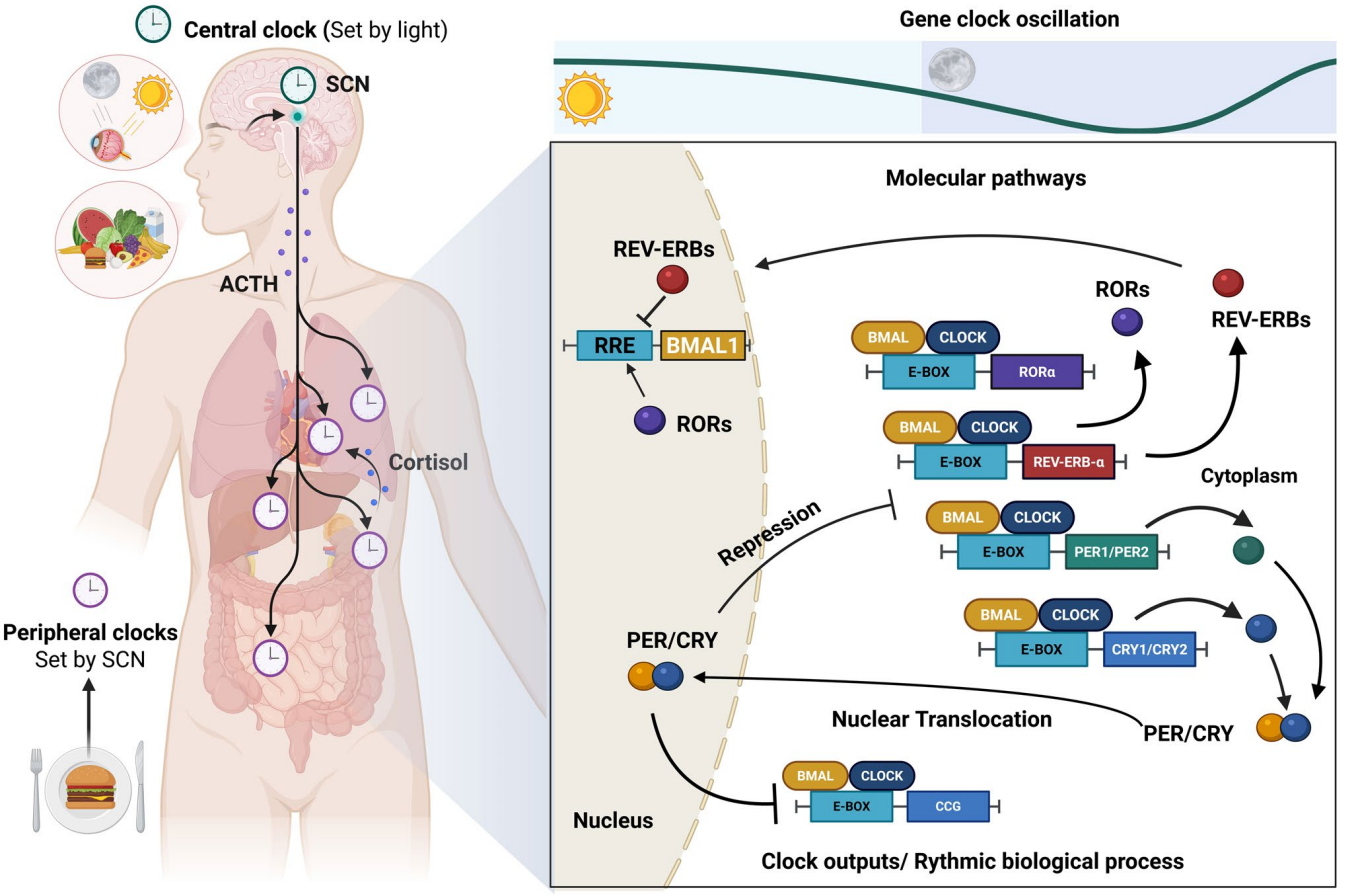
Entrainment and molecular regulation of circadian clocks. Circadian clocks are synchronized by external cues such as the light-dark cycle and feeding-fasting patterns, with the central clock in the suprachiasmatic nucleus (SCN) coordinating peripheral clocks located in peripheral organs. At the molecular level, circadian rhythms are generated by transcriptional-translational feedback loops involving core clock proteins that regulate rhythmic gene expression. ACTH: adrenocorticotropic hormone; BMAL1: Brain and Muscle ARNT-Like protein-1; CCG: clock-controlled genes; CLOCK: Circadian Locomotor Output Cycles Kaput; CRY: Cryptochrome protein; PER: Period protein; RRE: ROR response element. Created with *Biorender*

At the molecular level, both central and peripheral clocks rely on a cell-autonomous TTFLs mechanism involving the core clock genes and their respective proteins. Figure 1 illustrates the entrainment of central and peripheral clocks, driven primarily by light-dark cycle and external cues such as feeding-fasting patterns, along with the transcriptional-translational feedback mechanism underlying circadian rhythms.

The primary transcriptional activators include Circadian Locomotor Output Cycles Kaput (CLOCK), its paralogue Neuronal PAS Domains Protein 2 (NPAS2) and Brain and Muscle ARNT-Like protein-1 (BMAL1). These form heterodimers CLOCK-BMAL1 and NPAS2-BMAL1, whose peak expression in mammals occurs early in the morning, and that bind to E-box elements in the promoters of target genes to drive its transcription. These genes are Period (*Per1* and *Per2*) and Cryptochrome (*Cry1* and *Cry2*), whose transcripts accumulate during the afternoon [29].

Following translation, PER and CRY repressor proteins progressively accumulate in the cytoplasm throughout the late afternoon and evening, where they dimerize, and subsequently translocate into the nucleus. Once inside, they inhibit the transcriptional activity of the CLOCK: BMAL1 complex, thereby repressing their own expression [29].

As repression proceeds, *Per* and *Cry* gene expressions decline, as well as PER and CRY protein levels, due to ubiquitin-proteasome mediated degradation. This degradation relieves inhibition of the CLOCK-BMAL1 complex, allowing it to reinitiate its transcription the following morning, completing a new cycle. This delayed negative feedback generates oscillations with a period of nearly 24h, constituting the molecular basis of circadian rhythmicity [27].

Beyond regulating *Per* and *Cry*, the CLOCK-BMAL1 complex also induces the transcription of Nuclear Receptor Subfamily 1 Group D Member 1 (*Nr1d1)* and Member 2 *(Nr1d2)*, RAR-related Orphan Receptor alpha *(Rorα)* and beta (*Rorβ).* These genes encode auxiliary nuclear receptors REV-ERBα/β (NR1D1/NR1D2) and RORα/β, which act as stabilizing regulators of the circadian machinery. REV-ERBs and RORs exert opposing effects on *Bmal1* transcription, by competitively binding to ROR response elements (ROREs) within its promoter. RORs activate *Bmal1* transcription, whereas REV-ERBs repress it. This interlocked feedback loop confers additional robustness and stability to circadian oscillations [10].

### Mealtime as a Determinant Factor for Circadian Alignment and Metabolic Diseases

From an evolutionary perspective, organisms have developed metabolic adaptations that align EI with the highest metabolic demand periods, thereby optimizing nutrient absorption and energy storage during feeding, and energy mobilization during fasting [30]. The circadian system thus orchestrates energy homeostasis by temporally regulating hormonal secretion, neuronal signaling, and behaviors related to food intake and expenditure [30].

Food intake follows a circadian rhythm, regulated by a multi-oscillatory system that integrates signals from the central clock in the SCN with secondary food-entrainable clocks located in metabolic centers of the hypothalamus and the brainstem to coordinate the timing of food intake [30]. This “food clock” is account for driving anticipatory and behavioral responses to scheduled feeding, known as food-anticipatory activity (FAA) [30, 31]. In addition, peripheral clocks are entrained by food-derived signals, such as circulating nutrients and hormones, which convey energy status and nutrient availability to the brain, and contribute to feeding-related rhythms [30, 32].

Feeding, while under circadian control, also functions as a potent *zeitgeber*, particularly in metabolically active tissues such as the liver and pancreas. In this context, mealtime can shift the phase of peripheral circadian oscillators independently of the SCN, highlighting its critical role in circadian regulation [10, 33]. Evidence from animal and human studies shows that late-night eating or irregular feeding patterns disrupts the synchrony between central and peripheral clocks [8, 9, 33]. Such misalignment compromises metabolic coordination, leading to poor appetite regulation, weight gain, impaired glucose tolerance, dyslipidemia and systemic inflammation [10, 33–36].

In this context, TRE, which consolidates EI into a limited daily eating window aligned with the circadian rhythms, has been supported by the scientific community as a dietary approach to counteract metabolic disturbances caused by chrononutritional misalignment [10].

## A Brief Overview of TRE

### TRE: an Intermittent Fasting Approach

Fasting, defined as voluntary abstinence from foods and beverages for a specified period beyond the physiological overnight fast, has been practiced for millennia across civilizations for cultural, religious, ethical and health-related purposes [37]. Initially supported by empirical observations, this therapeutic use has, in recent decades, become the focus of scientific investigation. Evidence now links fasting to benefits such as improved metabolic regulation, enhanced insulin sensitivity, activation of autophagy and potential extension of lifespan. This has shifted its perception from a primarily spiritual or empirical practice to a potential evidence-based dietary strategy [38, 39].

In this context, intermittent fasting (IF) has attracted increasing interest as an alternative to continuous ER, with potential applications in obesity prevention and treatment [40]. Unlike ER, IF alternates periods of feeding and fasting, potentially conferring comparable metabolic benefits, although current evidence remains inconclusive [40, 41]. Among the IF protocols studied in humans, three approaches predominate: Alternate Day Fasting (ADF), the 5:2 Diet and TRE [41].

The ADF protocol alternates fasting days, either complete or limited to about 25% of daily energy needs, with days of unrestricted food intake [42]. The 5:2 diet consists of five days of unrestricted eating and two nonconsecutive or consecutive fasting days per week, usually with 500 and 1.000 kcal/day [43]. TRE, however, has gained prominence for its alignment of EI with circadian rhythms [41]. Table 1 summarizes the main characteristics, advantages, and limitations of the most common IF protocols.

**Table 1:** Comparison between the most common types of IF

Reference	IF protocol	Feeding period	Fasting period	Fasting frequency	Advantages	Limitations
Fanti et al. [41] Patterson and Sears [42] Varady et al. [43]	ADF	24h in alternated days	24h in alternated days	3–4 days per week	Modest weight loss Improvement in metabolic parameters	Extreme hunger on fasting days Low long-term adherence
Fanti et al. [41] Varady et al. [43]	5:2 Diet	5 days per week	2 consecutive or nonconsecutive days	2 days per week	Improvements in weight and mood Few negative side effects (e.g. irritable, low energy or hunger)	Modest or mixed effects in metabolic markers Metabolic benefits are not superior to ER
Fanti et al. [41] Patterson and Sears [42] Varady et al. [43]	TRE	4h per day 6h per day 8h per day 10h per day 12h per day 14h per day	20h per day 18h per day 16h per day 14h per day 12h per day 10h per day	7 days per week	Better adherence (especially for eating windows of ≥ 8h) Calorie counting is not required Alignment with the circadian cycle Improvement in cardiometabolic health parameters.	Methodological heterogeneity Scarcity of long-term evidence Social barriers

**ADF**: Alternate day fasting; **ER**: energy restriction; **IF**: intermittent fasting; **TRE**: Time-Restricted Eating

### TRE: The Human-adapted Version of TRF

TRE is an IF regimen in which food intake is confined to a consistent daily time window, typically ranging from 6 to 12h, without necessarily reducing total EI. Unlike other IF protocols that involve full-day fasting, TRE emphasizes daily rhythmicity and synchronization with the circadian clock, making it a potentially sustainable and physiologically aligned dietary strategy [41].

The concept TRE derives from TRF, a term originally applied in preclinical studies. A landmark investigation by Hatori et al. (2012) showed that mice fed a high-fat diet (HFD) within an 8h TRF window were protected from adverse diet-induced obesity and associated metabolic disturbances. In addition, these mice exhibited improved glucose tolerance and reduced hepatic steatosis, despite consuming similar calories as the ad libitum-fed control [44, 45].

Human studies evidence for TRE was first provided by Gill and Panda (2015). Their pilot study revealed that United States adults had an average eating window of 14–15h per day, which often extended on weekends due to social and lifestyle factors [46]. In the subsequent intervention phase, overweight individuals with baseline eating windows longer than 14h adopted a 10–11h TRE schedule and experienced weight loss, alongside improvements in sleep quality and overall well-being [46]. Since then, multiple studies have reported beneficial effects of TRE in diverse populations, ranging from healthy individuals to those with overweight, obesity, or metabolic disorders [15, 20, 47].

### Potential Mechanisms of TRE

**Fig. 2:**
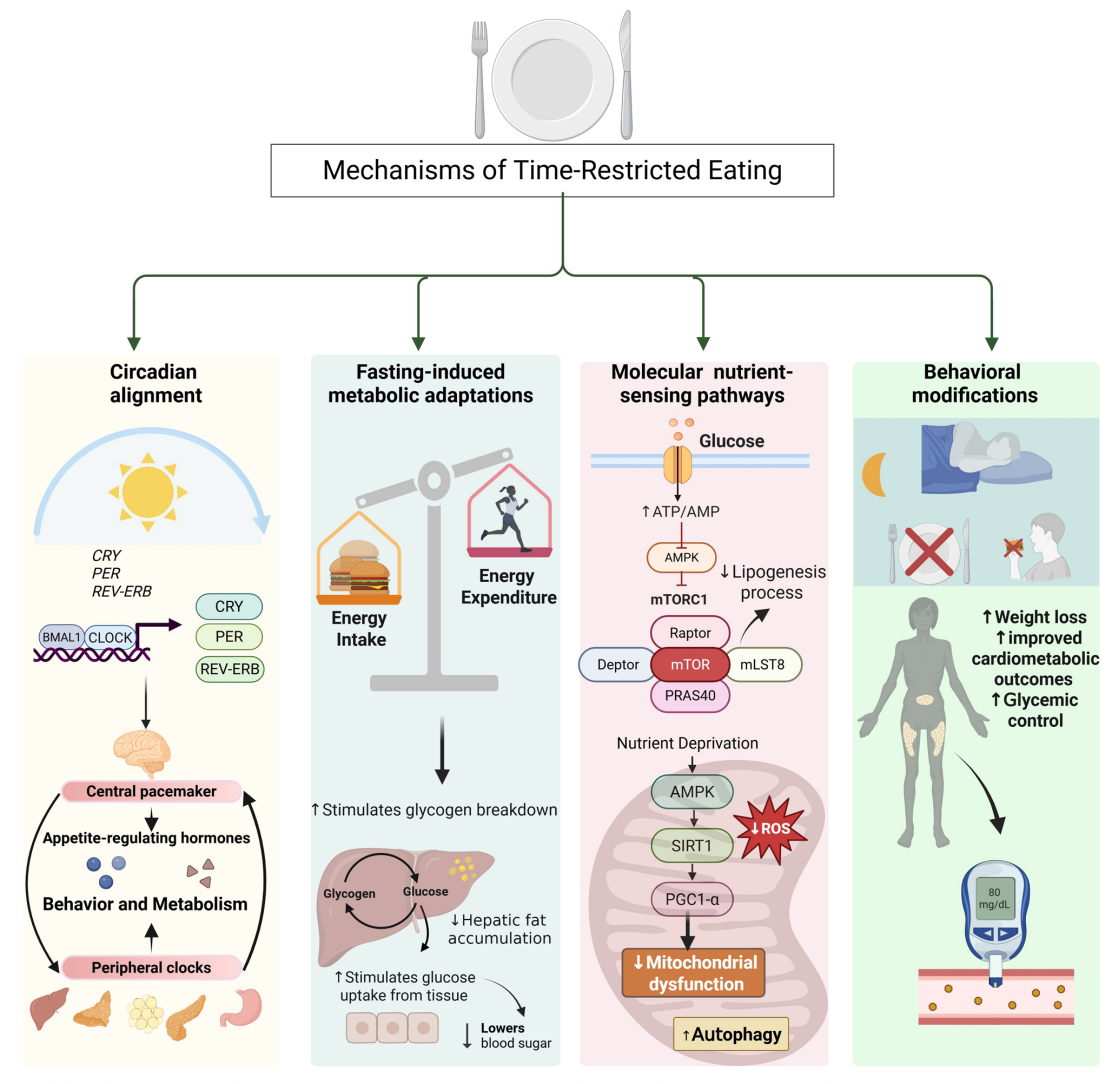
Potential mechanisms of Time-Restricted Eating. TRE appears to exert its metabolic benefits through the interaction between circadian alignment, fasting-induced metabolic adaptations, molecular regulation of nutrient-sensing pathways and beneficial feeding behavioral modifications. Together, these mechanisms contribute to improvements in body weight and cardiometabolic outcomes. AMP: adenosine monophosphate; AMPK: AMP-activated protein kinase; ATP: adenosine triphosphate; BMAL1: Brain and Muscle ARNT-Like protein-1; CLOCK: Circadian Locomotor Output Cycles Kaput; CRY: Cryptochrome protein; Deptor: DEP domain-containing mTOR-interacting protein; mLST8: mammalian lethal with Sect. 13 protein 8; mTOR: mechanistic target of rapamycin; mTORC1: mechanistic target of rapamycin complex 1; PER: Period protein; PGC1-α: Peroxisome proliferator-activated receptor gamma coactivator 1-alpha; PRAS40: Proline-rich Akt substrate of 40 kDa; Raptor: Regulatory associated protein of mTOR; ROS: Reactive oxygen species; SIRT1: sirtuin 1. Created with *Biorender*

Although advances have been made in the field of TRE, the physiological and molecular mechanisms underlying its metabolic effects remain incompletely understood. Current evidence indicates that the benefits of TRE likely arise from a multifactorial interplay between circadian alignment, extended fasting periods and behavioral modifications related to eating patterns [22]. Figure 2 illustrates the potential mechanisms through which TRE exerts its effects. A central mechanism proposed is the synchronization of food intake with the active phase of the circadian cycle. Key metabolic processes, including glucose and lipid metabolism, insulin sensitivity, mitochondrial function and secretion of appetite-regulating hormones, are regulated by circadian rhythms and operate more efficiently during the active phase of the day [13]. Aligning eating within this period may therefore optimize metabolic efficiency and energy balance [13]. Nonetheless, the direct effects of TRE on circadian systems in humans remain unclear, highlighting the need for further investigation [16, 17, 48].

Another important mechanism is the activation of fasting-associated metabolic pathways. Restricting food intake to a defined window prolongs fasting periods, which promotes the depletion of hepatic glycogen stores and a metabolic shift toward increased fatty acid oxidation, lipolysis and ketone body production, ultimately reducing hepatic fat accumulation [49]. In parallel, fasting activates nutrient-sensing pathways such as AMP-activated protein kinase (AMPK), while inhibiting the mechanistic target of rapamycin (mTOR), promoting metabolic efficiency, reducing oxidative stress, enhancing autophagy processes and improving insulin signaling [39, 45, 48, 50].

Emerging data suggest that fasting signals may also modulate peripheral circadian clocks via intracellular metabolic sensors such as AMPK and SIRT1. These sensors contribute to resetting the molecular clock by modulating transcription of core clock genes, which in turn govern the rhythmic expression of clock-controlled genes (CCGs) that regulate essential processes such as glucose and lipid metabolism and mitochondrial biogenesis [16].

Finally, behavioral modifications may further enhance TRE´s effects. Confining EI to a consistent window often eliminates late-night eating, a behavior strongly linked to weight gain and impaired glycemic control. Moreover, many individuals practicing TRE spontaneously reduce total EI without deliberate restriction, contributing to weight loss and improved cardiometabolic outcomes [20, 46]. While the precise contribution of each mechanism remains to be fully clarified, their combined effects likely underline the improvements reported in experimental and clinical studies.

## Cardiometabolic Outcomes of TRE Across Varying Durations of Eating Windows

TRE protocols are commonly categorized into three patterns: short (6h), moderate (8–10h), and long (12–14h) eating windows [51]. Each presents distinct advantages and limitations.

### Short Eating Windows

Short eating windows restrict EI to 4–6h with fasting periods of 18–20h per day. This is the most restrictive TRE approach and induces profound metabolic changes, including a shift from glucose to lipid utilization, increased fat oxidation, ketogenesis and stimulation of autophagy. These adaptations reduce insulin levels, improve mitochondrial function, attenuate chronic inflammation and promote favorable changes in body composition, particularly in individuals with overweight or obesity [39, 45, 51–54]. Despite these positive findings, they are derived from trials with small sample sizes and short intervention periods, making this TRE schedule the most frequently associated with adverse events and poorer long-term adherence due to its extensive fasting duration, which may compromise its feasibility and reproducibility [55].

In a randomized clinical trial, Cienfuegos et al. [54] showed that both 4h and 6h TRE led to similar reductions in body weight, insulin resistance and oxidative stress in comparison to the control group. All the results below were expressed as mean and [standard deviation]. For body weight, 4h and 6h of TRE produced greater reductions (both ∆ = -3.2% [0.4%]) in comparison with the control group (∆ = 0.1% [0.4%]). Fasting insulin (FI) was significantly decreased in both 4h and 6h of TRE (∆ = − 2.3 [1.5] mlU/mL and ∆ = -1.9 [1.1] mIU/mL, respectively) in comparison with controls (∆ = 3.5 [1.4] mIU/mL). Similarly, IR decreased by 29% and 12% in the 4h and 6h TRE groups, respectively. Regarding 8-isoprostane, a key marker of oxidative stress, 8 weeks of 4h and 6h TRE significantly reduced its levels (∆ = -13 [6] pg/mL and ∆ = -12 [4] pg/mL, respectively) compared with controls (∆ = 3 [3] pg/mL).

In the study conducted by Sutton et al. [13], eight men with prediabetes followed a 6h TRE protocol for five weeks, resulting in reductions in FI (-3.4 [1.6] mU/L), mean insulin (-26 [9] mU/L), and peak insulin (-35 [13] mU/L) levels, along with an increase in the insulinogenic index, a marker of β cell responsiveness (14 [7] U/mg). Regarding systolic and diastolic blood pressure (SBP and DBP, respectively), 5 weeks of 6h TRE protocol resulted in reductions for both parameters (-11 [4] and − 10 [4] mm, respectively), similarly to oxidative stress markers, in which 6h TRE group experienced a decrease of about 14% in comparison to controls.

Despite these promising results, extended fasting periods are often associated with a higher incidence of adverse events, including hunger, fatigue and irritability, as well as the difficulties in maintaining social eating habits, which all may limit long-term feasibility [55, 56]. Moreover, sustained adherence to more restrictive eating windows may increase the risk of nutritional inadequacy or disordered eating behaviors [55]. Consistent with this, Nie et al. (2023) reported no significant metabolic benefits were observed when comparing different eating window durations, suggesting that short eating windows are not metabolically superior to moderate eating windows.

### Moderate Eating Windows

Moderate eating windows, which encompass daily mealtime periods of 8–10h, have been suggested to be associated with greater sustainability, fewer adverse effects (e.g. irritability and fatigue) and improvements in mood [51, 57–60]. In this context, although much of available evidence derives from heterogeneous, short-term and limited sample-sizes studies, this strategy may represent a balance between metabolic efficiency and long-term adherence, and may therefore be considered the most pragmatic option between eating windows schedules.

In a 12 weeks study, Gabel et al. (2018) found that adults with obesity practicing an 8h TRE schedule experienced greater weight loss (-2,6% [5]) and reductions in SBP compared with the control group. Chow et al. [12] also reported an approximate 3% body weight reduction after 12 weeks of 8h TRE, with decreases in fat mass and visceral adiposity, however, these effects lost significance when adjusted for body weight loss. In adults with metabolic syndrome (MetS), a 10h TRE intervention for 12 weeks reduced body weight (-3.3 kg), body fat, LDL, cholesterol, and both SBP and DBP, while showing favorable trends in glycemic control [20].

In a randomized clinical trial, Wilkinson et al. [20] reported that adults with MetS who followed a 10 h TRE schedule for 12 weeks experienced reductions in body weight (-3.3 [3.2] kg), body fat (-1.01 [0.91] %), total cholesterol (-13.16 [24.29] mg/dL), low-density lipoprotein (LDL) (-11.94 [19.01] mg/dL), as well as SBP (-5.12 [9.51] mmHg) and DBP (-6.47 [7.94] mmHg). Favorable trends were also observed for fasting blood glucose (FBG, *p* = 0.081), FI (*p* = 0.064) and HbA1c (*p* = 0.058).

Moderate eating window aligns food intake with circadian metabolism, as most meals are consumed during the active phase of the day. This alignment enhances insulin sensitivity, improves lipid and glucose metabolism, and may reduce oxidative stress depending on dietary quality [19, 20, 61]. Furthermore, moderate eating periods often lead to reduced overall EI, contributing to improvements in body composition even without explicit ER [20, 57]. Nevertheless, Chaix et al. (2019) and Clavero-Jimeno et al. (2025) emphasize a need for studies that count for additional variables, such as meal timing, diet quality and sleep duration and quality, to fully establish the effectiveness of moderate TRE windows.

### Long Eating Windows

The long eating window is the most common dietary pattern, in which daily EI is spread over 12–14h. It is frequent among individuals with irregular routines, disorganized sleep habits, and unrestricted food access [46]. In fact, more than 50% of adults eat for 14.75h or longer per day, and this is often accompanied by a HFD, which is directly associated with health problems such as diabetes, cardiovascular diseases, hypertension and other preventable conditions [46].

Peeke et al.. (2021) compared the effects of 12h vs.14h of TRE in body weight and FBG of individuals with obesity. After 8 weeks, the least squares (LS) mean change in body weight was − 10.7 kg (8.5%) in the 14h group and − 8.9 kg (7.1%) in the 12h (both *p* < 0.001). The between-group LS mean difference was − 1.9 kg (1.4%), indicating a significantly greater reduction in the 14h group. For FBG, the LS mean change from baseline was 7.6 mg/dL in the 14h group (*p* < 0.05) and − 3.1 mg/dL in the 12h group, the latter not reaching statistical significance, with no significant difference between groups [62].

The long eating window often overlaps with circadian misalignment, as EI commonly occurs in the evening or night, periods characterized by reduced insulin sensitivity and metabolic efficiency, leading to impaired glycemic control and increased risk of obesity, IR and MetS [13, 63, 64]. Moreover, the absence of fasting periods can disrupt physiological processes such as autophagy, oxidative stress regulation and neuroendocrine balance. Although a long eating window is associated with higher adherence and consistency, which may improve mood, it may not provide significant health benefits [65].

In the general population, this pattern is highly prevalent and often linked to excess EI. However, when the quantity and quality of the food are controlled, it may still support an adequate diet and health [65]. Compared to this approach, moderate eating windows appear feasible and sustainable, promoting metabolic and cardiovascular benefits, particularly in glucose regulation, provided food quality is adequate [56]. Conversely, a short eating window extends fasting periods and may lead to rapid improvements in cardiometabolic markers and insulin sensitivity, but they are generally associated with poorer sleep and mood [55, 56]. The main characteristics and results of studies assessing cardiometabolic outcomes of varying eating windows in individuals with obesity included in this study are presented in Tables 2 and 3.

**Table 2:** Overall characteristics of studies assessing cardiometabolic outcomes of varying eating windows in individuals with obesity

Reference	Study design	Population	Sample size	Study duration	TRE protocol
Cienfuego et al. [54]	Parallel RCT	Adults with obesity	*n* = 49 *n* = 16 (4 h TRE) *n* = 19 (6 h TRE) *n* = 14 (control group)	8 weeks	Protocol 1: 4 h TRE with *ad libitum* feeding from 3 to 7 p.m. daily Protocol 2: 6 h TRE with *ad libitum* feeding from 1 to 7 p.m. daily
Sutton et al. [13]	RCT with crossover	Adult men with prediabetes and overweight or obesity	*n* = 8	5 weeks	6 h eTRE with dinner to be completed before 3 p.m.
Chow et al. [12]	RCT (feasibility)	Adults with obesity	*n* = 20 *n* = 11 (TRE group) *n* = 9 (control group)	12 weeks	8 h TRE with *ad libitum* feeding in a self-selected time frame
Lowe et al. [15]	RCT	Adults with overweight or obesity	*n* = 116 *n* = 59 (TRE group) *n* = 57 (control group)	12 weeks	8 h TRE with *ad libitum* feeding from 12 to 8 p.m.
Sampieri et al. [18]	Parallel RCT	Adults with normal weight or overweight	*n* = 32 *n* = 10 (control group) *n* = 8 (8 h TRE) *n* = 5 (10 h TRE) *n* = 9 (12 h TRE)	8 weeks	8 h TRE with *ad libitum* feeding from 10 a.m. to 6 p.m. 10 h TRE with *ad libitum* feeding from 9 a.m. to 7 p.m. 12 h TRE with *ad libitum* feeding from 8 a.m. to 8 p.m.
Manoogian et al. [61]	RCT	Adults with obesity and MetS	*n* = 108 *n* = 54 (TRE group) *n* = 54 (control group)	12 weeks	8–10 h TRE with *ad libitum* feeding in a self-selected time frame
Wilkinson et al. [20]	RCT	Adults with MetS	*n* = 19	12 weeks	10 h TRE with *ad libitum* feeding in a self-selected time frame
Jamshed et al. [66]	Parallel RCT	Adults with obesity	*n* = 90 *n* = 45 (control group + ER) *n* = 45 (eTRE group + ER)	14 weeks	8 h eTRE + ER from 7 a.m. and 3 p.m.

**Note:** included trials were predominantly of short duration (≤ 12 weeks). **ER**: energy restriction; **eTRE**: early time-restricted eating; **dTRE**: delayed time-restricted eating; **MetS**: metabolic syndrome; **sTRE**: self-selected time-restricted eating; **T2DM**: type 2 diabetes mellitus; **TRE**: time-restricted eating; **RCT**: randomized controlled trial

**Table 3:** List of major cardiometabolic outcomes of different eating windows from reviewed studies

Reference	Glycemic Outcomes		Lipid Profile		Blood Pressure		Oxidative / Inflammatory Markers	
	Evaluated parameters	TRE v Control *p* value	Evaluated parameters	TRE v Control *p* value	Evaluated parameters	TRE v Control *p* value	Evaluated parameters	TRE v Control *p* value
Cienfuegos et al. [54]^a^	↓ FBG ↓↓ FI* ↓↓ IR*	0.15 **0.02** **0.03**	↓ HDL ↑ LDL ↓ TG	0.93 0.76 0.96	↓ SBP ↓ DBP	0.06 0.11	↓ 8-IP* ↓ TNF-α ↑ IL-6	**0.02** 0.21 0.92
Cienfuegos et al. [54]^b^	↓ FBG ↓↓ FI* ↓↓ IR*	0.15 **0.04** **0.04**	↓ HDL ↓ LDL ↑ TG	0.93 0.76 0.96	↓ SBP ↓ DBP	0.06 0.11	↓ 8-IP* ↑ TNF-α ↑ IL-6	0.03 0.21 0.92
Sutton et al. [13]	↓ FBG ↓↓ FI* **↓**↓ IR*	0.49 **0.05** **0.005**	↓ HDL ↑ LDL ↑ TG*	0.48 0.75 **0.007**	↓ SBP* ↓ DBP*	**0.03** **0.03**	↓ 8-IP* ↑ IL-6	**0.05** 0.27
Chow et al. [13]	↓ FBG **↓** FI **↓** IR	0.98 0.84 0.95	**↑** HDL **↑** LDL **↓** TG	0.66 0.84 0.62	**↓** SBP **↓** DBP	0.78 0.77	NE	
Lowe et al. [15]	↓ FBG ↓ FI ↓ IR	0.50 0.60 0.42	↓ HDL **↑** LDL ↓ TG	0.42 0.62 0.29	**↓** SBP **↓** DBP	0.43 0.71	NE	
Sampieri et al. [18]	↓ FBG **↑** FI ↓ IR	0.30 0.16 0.20	↓ HDL ↓ LDL **↑** TG	0.08 0.37 0.78	NE		NE	
Manoogian et al. [61]	↓ FBG ↓ FI ↓ IR	-	↓ HDL ↓ LDL ↓ TG	-	↓ SBP ↓ DBP	-	NE	
Wilkinson et al. [20]	↓ FBG ↓ FI ↓ IR	0.08 0.06 0.10	**↓** HDL ↓↓ LDL* ↓ TG	0.051 **0.01** 0.84	**↓↓** SBP* **↓↓** DBP*	**0.04** **0.004**	NE	
Jamshed et al. [66]	**↓** FBG **↓** FI **↓** IR	0.53 0.20 0.23	**↓** HDL **↓** LDL **↓** TG	0.96 0.45 0.61	**↓** SBP ↓↓ DBP*	0.09 **0.04**	NE	

**Note:** included trials were predominantly of short duration (≤ 12 weeks). DBP: Diastolic blood pressure; FBG: Fasting blood glucose; FI: Fasting Insulin; HDL: High density lipoprotein; IL-6: Interleucin-6; IR: Insulin resistance; LDL: Low density lipoprotein; NE: Not evaluated; SBP: Systolic blood pressure; TG: Triglycerides; TNF-α: Tumor Necrosis Factor-alpha; TRE: Time-Restricted Eating; 8-IP: 8-isoprostane

↓: decrease in the mean value; ↓↓: statistically significant decrease; ↑: increase in the mean value; ↑↑: statistically significant increase

*: p value less than 0.05

a: results from the 4-hour TRE group

b: Results from the 6-hour TRE group

## Durations of Eating Windows and Timing: Which Is the Most Determinant for Improved Cardiometabolic Outcomes?

Studies on TRE field generally assess the effects of this dietary approach according to the duration and timing of eating windows. Regarding duration, the most frequently evaluated windows in both clinical and experimental studies range from 4 to 1h. Among these, the 8h eating window followed by a 16h fasting period (usually referred to as the 16:8 protocol) has been the most extensively studied and widely adopted due to its balance between feasibility, metabolic efficacy and adherence potential, although there is no consensus regarding the optimal eating window [57].

Regarding the timing of eating windows, TRE can be classified into three main approaches: early Time-Restricted Eating (eTRE), in which eating typically begins at or before 8:00 a.m.; mid or standard TRE, with eating windows beginning between 10 a.m. and 12 p.m.; and delayed Time-Restricted Eating (dTRE) or late Time-Restricted Eating (lTRE), where eating starts between 12 p.m. and 1 p.m. and extends into the evening [1, 67]. Some protocols prescribe fixed timings of eating windows to standardize intervention, whereas others allow participants to choose their eating period, referred as self-selected TRE [68].

Currently, there is also no consensus on the optimal TRE timing. Evidence suggests that eTRE offers greater metabolic benefits, particularly in glucose metabolism and insulin sensitivity, compared with no TRE, whereas lTRE does not consistently produce these benefits, although these approaches may improve long-term adherence [69–71]. Similarly, when combined with ER, eTRE promotes greater improvements in anthropometric and metabolic parameters compared to lTRE + ER or ER alone [72, 73]. However, the limited differentiation observed between TRE modalities in current studies may be attributed to the relative short duration of most interventions (typically 8 to 12 weeks) and the lack of fasting prescriptions tailored to individual chronotype. Longer trials are likely required to reveal more pronounced metabolic changes consistent with chrononutrition principles.

It remains unclear whether cardiometabolic improvements associated with TRE interventions are primarily driven by timing or duration of eating windows. Timing acts primarily as a circadian modulator, enabling food intake to be aligned with endogenous circadian rhythms, which has been suggested to play a critical role in metabolic regulation, particularly given recent associations between late-night eating and poorer metabolic health [46, 74]. In contrast, the effects related to the duration of eating windows seem to be largely mediated by the metabolic stimulus induced by longer fasting periods, along with reduction in EI conferred by restricting food intake to a fixed period of hours per day [75].

Current evidence suggests that these mechanisms are partially independent yet interactive, such that health benefits of TRE may arise from the combination between metabolic stimulus and energy deficit provided by duration and circadian alignment conferred by timing. Importantly, the available literature does not allow the establishment of a clear hierarchy between these factors in determining cardiometabolic outcomes [76]. To date, no study has directly compared the relative contributions of duration versus timing of eating windows on cardiometabolic outcomes of individuals with obesity, underscoring the need for future research to clarify what is essential for optimizing metabolic benefits: timing or duration isolated or the combination of both [51, 68, 77].

## Challenges, Limitations and Future Perspectives

One of the main challenges in TRE studies relates to social barriers. Difficulties in adhering to TRE protocols during social events or weekends, when individuals often change their dietary and physical activity patterns, along with disapproval from peers or family remain significant obstacles to implementation [78, 79]. These barriers highlight the importance of a supportive social environment and reinforce the need for personalized strategies to improve both feasibility and effectiveness [79]. Understanding the multilevel factors that influence adherence to TRE, such as social support, flexibility, and environmental influences, is crucial for promoting TRE long-term use and sustainability.

Methodological limitations also restrict current evidence. Most studies available both in literature and in this review are characterized by short intervention periods (4–12 weeks) and small sample sizes with a homogenous population, which limit the reliability and generalizability of findings. These constraints limit firm conclusions, particularly across diverse populations and in long-term settings. Future research should prioritize well-designed, long-term randomized clinical trials with larger and heterogeneous samples, assessing not only metabolic outcomes but also adherence, feasibility and real-world applicability to support TRE as a sustainable dietary strategy [68, 71, 80, 81]. Another important limitation concerns dietary control. Although TRE has been shown to improve metabolic parameters, it remains unclear whether these effects are attributable to TRE itself or simply to reduced EI resulting from a shorter eating window [82].

An important question remaining in literature is whether the optimal timing of eating window is determinant for optimizing outcomes, in which several studies have shown that individuals adhering to eTRE achieve superior cardiometabolic improvements compared with those following lTRE or dTRE [66, 83]. Given that timing of eating windows acts as a circadian modulator, it is important to consider individual circadian preferences, typically assessed using chronotype questionnaires. In this context, emerging evidence from the field of chrononutrition suggests that synchronizing eating windows with an individual’s chronotype may enhance the benefits of TRE, although findings remain mixed and inconclusive [84, 85]. Nevertheless, most randomized clinical trials (RCTs) have not yet tailored TRE protocols to participants’circadian preferences. Incorporating chronotype-based approaches in future personalized TRE interventions could uncover new insights into metabolic regulation and addressing this gap may represent a promising direction for optimizing the clinical applicability and outcomes of TRE [86].

## Conclusion

In conclusion, TRE shows strong potential as a lifestyle-based strategy for enhancing cardiometabolic well-being, particularly among people living with overweight and obesity. Although moderate eating windows of 8–10h currently appear to represent the most pragmatic option due to its balance between metabolic benefits and long-term adherence, these findings are derived from heterogeneous, short-term and limited sample-sizes studies. Therefore, no consensus has yet been reached regarding the optimal duration of the eating windows in TRE interventions for individuals with obesity. Thus, further research is needed to establish the most effective duration to maximize cardiometabolic benefits in individuals, as well as if TRE strategies should be personalized according to individual’s chronotype and lifestyle to promote adherence and superior clinical outcomes.

## Key References

- Schrader LA, Ronnekleiv-Kelly SM, Hogenesch JB, Bradfield CA, Malecki KM. Circadian disruption, clock genes, and metabolic health. J Clin Invest. 2024;134(14):e170998. Published 2024 Jul 15. doi:10.1172/JCI170998
  - ○ This review provides a comprehensive analysis of how circadian disruption and altered expression of core clock genes contribute to metabolic dysregulation. The findings highlight that synchronizing food intake with endogenous circadian rhythms may be a key strategy to improve metabolic health.
- Petersen MC, Gallop MR, Flores Ramos S, Zarrinpar A, Broussard JL, Chondronikola M, Chaix A, Klein S. Complex physiology and clinical implications of time-restricted eating. Physiol Rev. 2022 Oct 1;102(4):1991-2034. doi: 10.1152/physrev.00006.2022. Epub 2022 Jul 14. PMID: 35834774; PMCID: PMC9423781.
  - ○ This comprehensive review integrates molecular, physiological, and clinical perspectives on Time-Restricted Eating, discussing the mechanisms through which fasting-feeding cycles regulate circadian metabolism and energy homeostasis. It synthesizes evidence from both preclinical and human studies, identifying critical gaps and outlining directions for future research in chrononutrition.
- Ezpeleta M, Cienfuegos S, Lin S, Pavlou V, Gabel K, Tussing-Humphreys L, Varady KA. Time-restricted eating: Watching the clock to treat obesity. Cell Metab. 2024 Feb 6;36(2):301-314. doi: 10.1016/j.cmet.2023.12.004. Epub 2024 Jan 3. PMID: 38176412; PMCID: PMC11221496.
  - ○ This recent review summarizes the latest randomized controlled trials assessing the effects of Time-Restricted Eating on body weight, metabolic risk factors, and cardiometabolic health in individuals with obesity. The authors critically evaluate the variability of eating windows across studies and discuss factors influencing adherence and long-term efficacy.

## Data Availability

No datasets were generated or analysed during the current study.

## Abbreviations

ADF: alternate day fasting
AMPK: AMP-activated protein kinase
BMAL1: brain and muscle ARNT-like protein-1
CCG: clock-controlled genes
CLOCK: circadian locomotor output cycles kaput
CRY: cryptochrome proteins
Cry: cryptochrome genes
dTRE: delayed time-restricted eating
ER: energy restriction
EI: energy intake
eTRE: early time-restricted eating
FAA: food-anticipatory activity
FBG: fasting blood glucose
FI: fasting insulin
HDL: high-density lipoprotein
HFD: high-fat diet
IF: intermittent fasting
LDL: low-density lipoprotein
lTRE: late time-restricted eating
mTOR: mechanistic target of rapamycin
MetS: metabolic syndrome
NPAS2: neuronal PAS domains protein 2
NR1D1: nuclear receptor subfamily 1 group D member 1
NR1D2: nuclear receptor subfamily 1 group D member 2
PER: period protein
Per: period gene
pRGCs: photosensitive retinal ganglion cells
RHT: retinohypothalamic tract
ROR: RAR-related orphan receptor
ROREs: ROR response elements
SCN: suprachiasmatic nucleus
SIRT1: sirtuin 1
TRE: time-restricted eating
TRF: time-restricted feeding
TTFLs: transcription–translation feedback loops
WHO: World Health Organization
